# Risk factors for long-term invasive mechanical ventilation: a longitudinal study using German health claims data

**DOI:** 10.1186/s12931-024-02693-6

**Published:** 2024-01-27

**Authors:** Franziska C. Trudzinski, Julia D. Michels-Zetsche, Benjamin Neetz, Jan Meis, Michael Müller, Axel Kempa, Claus Neurohr, Armin Schneider, Felix J. F. Herth, Joachim Szecsenyi, Elena Biehler, Thomas Fleischauer, Michel Wensing, Simone Britsch, Janina Schubert-Haack, Thomas Grobe, Timm Frerk, The PRiVENT-study group, Felix Herth, Gabriele Iberl, Julia Dorothea Michels, Beatrice Müller, Michael Müller, Benjamin Neetz, Franziska Christina Trudzinski, Martina Bentner, Elena Biehler, Thomas Fleischhhauer, Johanna Forstner, Gerhard Fuchs, Nicola Litke, Markus Qreini, Selina von Schumann, Noemi Sturm, Joachim Szecsenyi, Aline Weis, Michel Wensing, Timm Frerk, Thomas Grobe, Janina Schubert-Haack, Anja Klingenberg, Jan Meis, Alex Kempa, Biljana Joves, Andreas Rheinhold, Ahmed Ehab, Claus Neurohr, Alessandro Ghiani, Nina Lutz, Swenja Walcher, Konstantinos Tsitouras, Joanna Paderewska, Selina Briese, Armin Schneider, Christoph Rauch, Patrick Gehrig, Joachim Sugg, Susanne Hirschmann, Simone Britsch, Christa Straub, Claude Jabbour, Michael Hahn, Jörg Krebs, Peter-Tobias Graf, Petra Denzer, Uta Merle, Mascha Fiedler, Guido Hundt, Jens Regula, Miriane Bomeken, Sebastian Stier, Jens Müller, Ute Oltmanns, Tom Terboven, Marcus Hennersdorf, Neslihan Satir, Mathias Borst, Brigitte Mayer, Wolfgang Reikow, Markus Kredel, Patrick Keppeler, Konstantin Frey, Holger Wolff, Florian Seidlitz, Stefanie Bientzle, Boris Nohé, Sebastian Allgäuer, Alexej Schöpp, Christoph Schlegel, Imke Hübner, Andrezj Kuzniar, Helene Häberle, Reimer Riessen, Benjamin Schempf, Ingo Rebenschütz, Andreas Straub, Marc Kollum, Markus Winter, Paul Hartveg, Andreas Junginger, Helmut Beck, Mathias Vogel, Ralf Völker, Thomas Wiesmann

**Affiliations:** 1 Department of Pneumology and Critical Care, Thoraxklinik Heidelberg gGmbH, Heidelberg, Translational Lung Research Center Heidelberg (TLRC-H), German Center for Lung Research (DZL), Heidelberg, Germany; 2 Institute of Medical Biometry, University of Heidelberg, Heidelberg, Germany; 3 Department of Pneumology and Critical Care, SLK-Klinik Löwenstein, Löwenstein, Germany; 4 Department of Pneumology and Respiratory Medicine, Robert-Bosch-Krankenhaus Klinik Schillerhöhe, Gerlingen, Germany; 5 Department of Anesthesia and Intensive Care Medicine Waldburg-Zeil Kliniken, Wangen Im Allgäu, Germany; 6 Department of General Practice and Health Services Research, University Hospital Heidelberg, Heidelberg, Germany; 7 aQua Institute for Applied Quality Improvement and Research in Health Care, Göttingen, Germany; 8 Department of Cardiology, Angiology, Haemostaseology and Medical Intensive Care, University Medical Center Mannheim, Mannheim, Germany; 9 European Center for Angioscience (ECAS) and German Center for Cardiovascular Research (DZHK), Partner Site Heidelberg/Mannheim, Mannheim, Germany

**Keywords:** Long-term invasive mechanical ventilation, Invasive home mechanical ventilation, Weaning, Weaning failure, Prolonged weaning, Prognostic factors, Predictive model

## Abstract

**Background:**

Long-term invasive mechanical ventilation (IMV) is a major burden for those affected and causes high costs for the health care system. Early risk assessment is a prerequisite for the best possible support of high-risk patients during the weaning process. We aimed to identify risk factors for long-term IMV within 96 h (h) after the onset of IMV.

**Methods:**

The analysis was based on data from one of Germany's largest statutory health insurance funds; patients who received IMV ≥ 96 h and were admitted in January 2015 at the earliest and discharged in December 2017 at the latest were analysed. OPS and ICD codes of IMV patients were considered, including the 365 days before intubation and 30 days after discharge. Long-term IMV was defined as evidence of invasive home mechanical ventilation (HMV), IMV ≥ 500 h, or readmission with (re)prolonged ventilation.

**Results:**

In the analysis of 7758 hospitalisations, criteria for long-term IMV were met in 38.3% of cases, of which 13.9% had evidence of HMV, 73.1% received IMV ≥ 500 h and/or 40.3% were re-hospitalised with IMV. Several independent risk factors were identified (p < 0.005 each), including pre-diagnoses such as pneumothorax (OR 2.10), acute pancreatitis (OR 2.64), eating disorders (OR 1.99) or rheumatic mitral valve disease (OR 1.89). Among ICU admissions, previous dependence on an aspirator or respirator (OR 5.13), and previous tracheostomy (OR 2.17) were particularly important, while neurosurgery (OR 2.61), early tracheostomy (OR 3.97) and treatment for severe respiratory failure such as positioning treatment (OR 2.31) and extracorporeal lung support (OR 1.80) were relevant procedures in the first 96 h after intubation.

**Conclusion:**

This comprehensive analysis of health claims has identified several risk factors for the risk of long-term ventilation. In addition to the known clinical risks, the information obtained may help to identify patients at risk at an early stage.

*Trial registration* The PRiVENT study was retrospectively registered at ClinicalTrials.gov (NCT05260853). Registered at March 2, 2022.

**Supplementary Information:**

The online version contains supplementary material available at 10.1186/s12931-024-02693-6.

## Background

The increasing number of patients who cannot be successfully weaned from invasive mechanical ventilation (IMV) after an acute stay in the intensive care unit (ICU) is a drawback of modern intensive care medicine (ICM) [1]. Providing outpatient care for patients receiving IMV is time-consuming, expensive and also takes up trained staff who are urgently needed to provide inpatient care. Even patients at high risk of weaning failure can often be successfully weaned from IMV in specialist weaning centres [2]. However, transfer to long-term IMV facilities after weaning failure often occurs directly from the ICU, without prior assessment in one of these centres. Even when a patient is transferred to a weaning centre, it is often after a long inpatient history of IMV. Unfavourably, duration of previous IMV is an independent risk factor for weaning failure with subsequent discharge to invasive home ventilation [3–5]. In addition to long waiting lists and limited capacity in specialised centres, these individuals are critically ill patients with difficult to predict disease trajectories, making anticipatory care planning challenging. The number of acute or post-operative invasively ventilated patients who require prolonged weaning is costly and consumes valuable critical care resources. The recent international, multicentre, observational study WEAN SAFE with 5869 critically ill adult patients, shows that only 65% of patients who received invasive ventilation for more than 2 days were successfully weaned on day 90 [6]. It is estimated that weaning accounts for approximately 40% of total ventilation time, mainly due to patients requiring prolonged weaning [7]. The group of previously identified risk factors for prolonged mechanical ventilation (PMV) is heterogeneous and includes comorbidities, site of intubation, various laboratory or blood gas parameters, ventilator settings, functional parameters and intensive care scoring systems. Identified risks of weaning failure are mainly related to age, delayed initiation of weaning, higher sedation scores, previous home mechanical ventilation, cause of ventilation and also pre-existing underlying diseases. Elevated PaCO_2_ levels during spontaneous breathing trials indicate both prolonged weaning and weaning failure [6, 8]. Patients with an increased risk of long-term ventilation and weaning failure should ideally receive specific support throughout the course of IMV. To achieve this, reliable tools are needed to assess the risk of long-term IMV as early as possible during the ICU stay. Comprehensive data on this important issue is needed to realistically map all the different risks in this large group of ventilated patients from different intensive care units. The aim of our study was to analyse the risk factors for long-term IMV early in the course of intensive care treatment. We used data from one of the largest statutory health insurance funds in Germany to analyse a large population of ventilated patients. In a second step, a prognostic model was to be created based on the identified risk factors, which enables an estimation of the risk of long-term ventilation at an early stage after intubation. This work is part of the multicentre PRiVENT study project, which aims to investigate innovative forms of care for invasively ventilated patients [9].

## Methods

The present study applied the STROBE (Strengthening the Reporting of Observational Studies in Epidemiology) guidelines. The basis for the analysis is the claims data of *Allgemeine Ortskrankenkasse Baden-Württemberg* (AOK-BW), one of the largest nationwide health insurance companies with around 4.38 million insured people, which corresponds to around 5.96% of the population covered by statutory health insurance in Germany. That is about 43% of the population of the state of Baden-Würrtemberg whose population equals that of Belgium and is larger than Denmark or Norway. The validity follows to the requirements of German health claims data. The data were provided in pseudonymised form and analysed by the Institute for Applied Quality Improvement and Research in Health Care, aQua, in close exchange with a team of experienced clinicians. The consulting team consisted of 3 specialists in pneumology and internal medicine with additional qualifications in intensive care medicine, and a respiratory therapist.

### Patients

The patients studied were AOK-BW insured patients, who underwent invasive mechanical ventilation during a hospital stay with earliest admission date in January 2015 and latest discharge in December 2017. To specifically identify high-risk patients, only patients who were invasively ventilated for ≥ 96 h, were over 30 years of age, and had a medical comorbidity were included. Patients with evidence of previous invasive HMV, or neuromuscular disease without potential for ventilator weaning, were excluded from the analysis, as it is clear, that these patients are already at high risk for long-term IMV. Patients who died in the first 11 days were also excluded from the analyses. We focussed on those who survived this first critical phase in order to study a population where the risk of long-term ventilation is a relevant consideration. In order to capture pre-existing diagnoses and chronic conditions and to document the sustainability of weaning from ventilation, the patient had to be insured with AOK-BW within the previous 365 days and 30 days after discharge from the hospital. The inclusion and exclusion criteria as well as their definitions are shown in Table 1.

**Table 1:** Selection criteria

	Definition
*Inclusion criteria*	
At least 96 h of ventilation	Ventilation hours ≥ 96 (counting method according to the German Coding Guidelines)
At least 30 years old	Start of inpatient treatment [year]—Year of birth ≥ 30
At least one comorbidity	One of the following ICDs coded in the 365 days prior to the ventilation case: J44, M41, J60-J70, J84, I50, I25, E10-E14, E66.01, E66.02, C00-C97, F05, F10.4–16.4 (in each case those ending in .4), F18.4, F19.4, F20-29, G62.80, G72.80, N17, N18
Insurance periods	In calendar years in which the pre-review period (365 days before the start of the inpatient stay), the ventilation case and the post-review period (30 days after discharge) fall, the insured person must have been insured with AOK-BW for at least 365 days
*Exclusion criteria*	
Neuromuscular diseases	Exclusion of insureds with a coded condition with ICD G12.2 and/or G71 within 365 days prior to the ventilator case
No prior invasive HMV	OPS 8716.01, 8716.11, 8716.21, „Tracheostomy ventilator aids “12.50.99.0002, ICD (Z99.1, Z43.0, ICD Z99.1) within 365 days prior to the ventilator case
No death within 11 days following the initiation of ventilation	No death (discharge reason ≠ death) within 11 days of the first date on which access for invasive ventilation was coded (OPS 5311, 5312, 8701, 8704)

The table shows the inclusion and exclusion criteria of the study, and the respective definitions of the given parameters

*HMV* invasive mechanical home ventilation, *ICD* international statistical classification of diseases and related health problems, *OPS* official classification of operational procedures in Germany

### Outcomes

Long-term IMV was defined as follows; evidence of invasive home mechanical ventilation after discharge, or total duration of ventilation ≥ 500 h, or re-hospitalisation with (re)prolonged ventilation (IMV ≥ 96 h). The criteria and operationalisations of outcomes are listed in Table 2.

**Table 2:** Definition of outcomes

Criteria	Definition
*Evidence of home invasive ventilation after discharge*	
Initiation of home mechanical ventilation - Invasive HMV after weaning failure and within 30 days	OPS 8716.01
Control or optimisation of a previously initiated HMV within 30 days	OPS 8716.11
Termination of previously initiated home ventilation within 30 days	OPS 8716.21
Tracheostomy ventilator aids prescribed after start of ventilation and within 30 days of discharge	Nr. 12.50.99.0002*
Inpatient: dependence (long term) on respirator after start of ventilation AND Care of a tracheostoma after the start of ventilation and within 30 days after discharge	ICD Z99.1
	ICD Z43.0
Outpatient: Dependence (long-term) on respirator AND Care of a tracheostoma in the quarter following the end of the respirator claim	ICD Z99.1
	ICD Z43.0
*Total duration of ventilation ≥ 500 h*	
Total duration of ventilation is 500 or more hours	Ventilation hours ≥ 500
*Re-hospitalisation with (re)prolonged ventilation*	
Re-hospitalisation with (re)prolonged ventilation within 30 days after discharge	Re-hospitalisation with initiation of prolonged ventilation within 30 days of discharge (with ventilation hours ≥ 96)

The table shows the different definitions of the three outcomes studied; long-term IMV defined as evidence of invasive mechanical home ventilation, IMV ≥ 500 h and/or readmission with (re)prolonged ventilation

*HMV* invasive mechanical home ventilation, *IMV* invasive mechanical ventilation, *ICD* international statistical classification of diseases and related health problems, *OPS* official classification of operational procedures in Germany. *The specified no. 12.50.99.0002 is an AOK-BW specific code

### Analysis of claims data

**Fig. 1:**
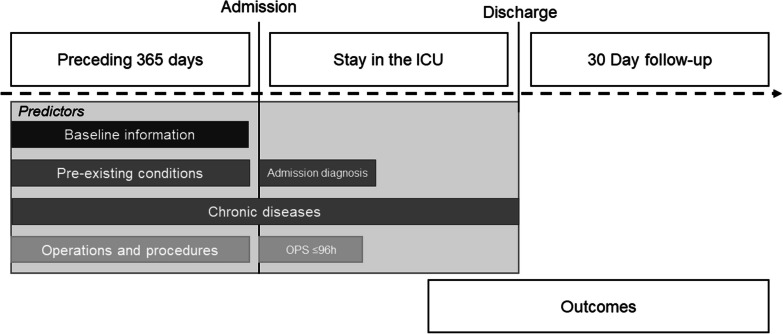
Overview of the time periods of predictors and outcomes. The analysis was based on data from the AOK Baden-Württemberg; patients who received IMV ≥ 96 h and were discharged between 2015 and 2017 were analysed. Health claims data were considered, in each case for the previous year and 30 days after hospitalisation. Abbreviations: OPS official classification of operational procedures in Germany

The data was analysed in a multi-stage process. First, (a) a systematic literature review on risk factors for long-term ventilation in hospitalised patients was performed [8]. With temporal overlap "historical" statutory health insurances routine data were analysed to exploratively identify (b) characteristics of hospitalised patients, which in bivariate evaluations are empirically associated with an increased risk for invasive long-term ventilation. Thus, all 3-digit ICD (International Statistical Classification of Diseases and Related Health Problems) codes (and selected 4-digit codes) and 4-digit official classification of operational procedures in Germany (OPS) codes (and selected 5-digit codes) were processed for different time periods. The observed diagnosis or OPS relative frequencies of patients with long-term ventilation were related to the expected relative frequency based on the total population. If the number of patients on long-term ventilation differed significantly from the total population, this characteristic was considered in further analysis steps. Only complete cases were used for further analysis (complete-case analysis). Subsequently, (c) the correspondingly identified potential predictors were tested with regard to their statistically independent influences on the risk for invasive long-term ventilation in logistic regression models. Data from ICD codes, OPS, and prescriptions for medical aids were considered. As the OPS code in intensive care stays are coded on a daily basis, we were able to selectively consider the procedures of the first 96 h in the analyses. The period 0–24 h applies here for intubation as well as for the other OPS codes. For the analyses, the day of intubation was defined as day 0. In addition to the data on the hospitalisation at which the invasive ventilation was initiated, information on the previous year and the subsequent 30 days of the corresponding hospitalisation were also considered. Predictors were selected stepwise based on exploratory data analysis in close communication with the consulting team. All relevant independent predictors were combined in a final regression model. Multicollinearity was assessed by examining tolerance and the variance inflation factor (VIF). No predictor had to be excluded in the final model. An overview of the time periods of the predictors and results is shown in Fig. 1.

### Statistical methods

Binary logistic regression models were estimated to predict the risk for long-term IMV. To investigate the predictive value of the final model, the probabilities determined in regression models were evaluated by Receiver Operating Characteristic (ROC) analyses. The performance of the model was evaluated on a 2018 AOK data-set (which was not part of the data used for creating the model). Statistical analyses were performed using SAS Enterprise Guide 7.1.

## Results

**Fig. 2:**
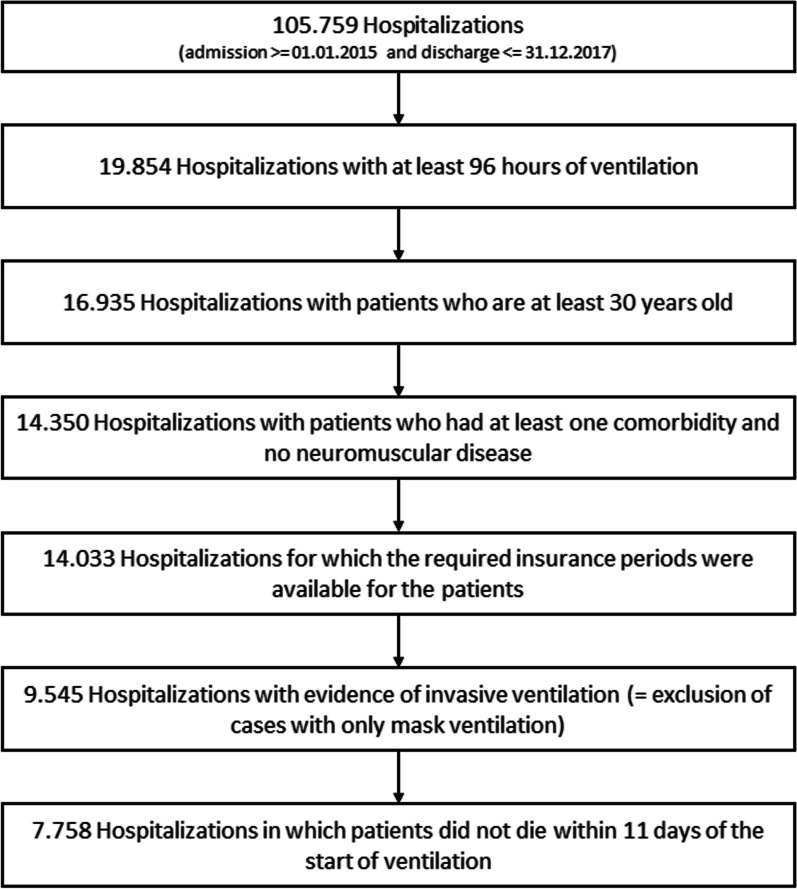
Consort diagram of inclusions and exclusions

Out of a total of 105,759 hospitalisations during the study period, 7758 that met the inclusion criteria were included in the final analyses. A flow chart with all patients and the respective inclusion and exclusion criteria is depicted in Fig. 2**.** In the studied patient population, the proportion of female patients was 37.2%, the in-hospital mortality rate was 25.2% and the mean duration of mechanical ventilation was 429.6 h. By the end of the 30-day follow-up, the criteria for long-term IMV were met in 2905 of 7758 hospitalisations (38.3%). Of the 2905 hospitalisations, 13.9% showed evidence of invasive HMV, 73.1% were ventilated for at least 500 h and/or 40.3% of patients hospitalised were re-hospitalised receiving IMV within the follow-up period of 30 days after discharge.

### Regression analyses

**Fig. 3:**
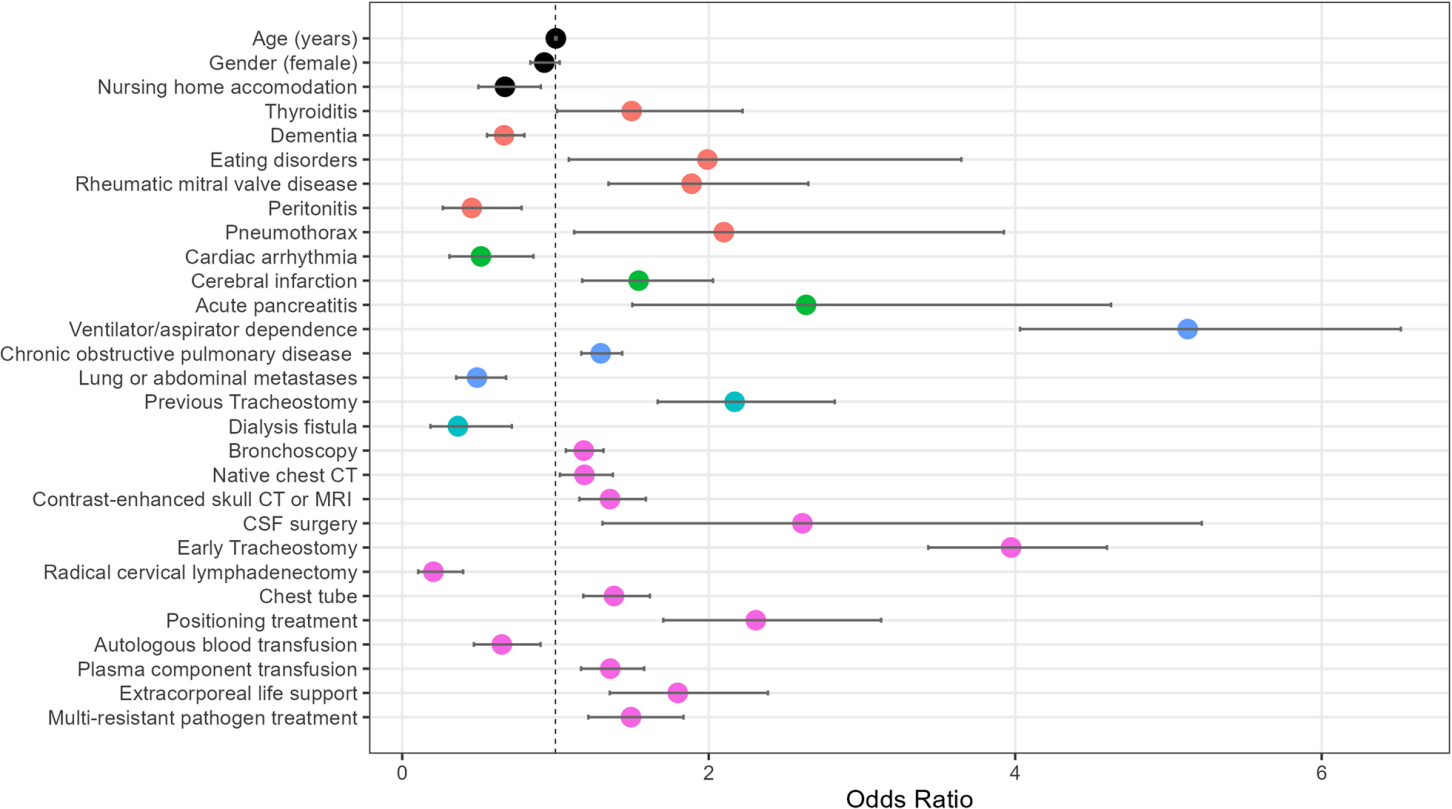
Identified risk factors for long-term invasive mechanical ventilation. The figure shows all predictors of the model with their respective odds ratios and confidence intervals. In addition to stem data (black dots), pre-existing conditions coded in the 365 days prior to the index case (red dots), admission diagnosis (green dots), pre-existing conditions, admission diagnosis in the last 365 days (blue dots), operations and procedures prior to the index stay from the same period (turquoise dots) and operations and procedures during the hospital stay up to 95 h after intubation (pink dots) were considered. *COPD* chronic obstructive pulmonary disease, *CSF* cerebrospinal fluid, *CT* computed tomography, *MRI* magnetic resonance imaging. Extracorporeal life support includes pumpless extracorporeal lung assist, extracorporeal CO2 removal, veno-venous and veno-arterial, ECMO extracorporeal membrane oxygenation

Among the baseline predictors, only nursing home placement immediately prior to hospitalisation was relevant, and this was associated with a favourable prognosis; age or gender did not play a role. In terms of pre-existing conditions documented in the year preceding the corresponding hospitalisation, and/or chronic diseases, conditions recorded during the hospital stay, thyroiditis, eating disorders, rheumatic mitral valve disease (insufficiency or stenosis), pneumothorax and chronic obstructive pulmonary disease (COPD) as well as dependence (at least 3 completed months) on aspirator and/or respirator carried an increased risk of long-term IMV whereas a prior diagnosis of dementia or peritonitis showed a favourable prognosis with respect to the risk of long-term IMV. Regarding the admission diagnoses, cardiac arrhythmias were associated with a favourable prognosis while cerebral infarction and acute pancreatitis entailed an increased risk for long-term IMV. In the analysis of the operations and procedures documented in the preceding year of the corresponding hospitalisation, a previous tracheostomy was particularly unfavourable, whereas the application of a dialysis shunt was associated with a lower risk of long-term IMV. Surgeries and procedures associated with increased risk of long-term IMV during the first four days of IMV included bronchoscopies, computed tomography of the chest, cranial magnetic resonance imaging, cerebrospinal fluid system procedures (drainage, shunt, catheterisation), positioning treatment in a special bed, the transfusion of plasma components, the use of extracorporeal life support (PECLA, ECCO_2_R, vv- and va-ECMO and pre-ECMO therapy) and the complex treatment of colonisation or infection with multidrug-resistant pathogens. Radical cervical lymphadenectomy and autologous blood collection and transfusion showed favourable prognosis in terms regarding outcome. All investigated predictors with the corresponding odds ratios and confidence intervals (CI) are shown in Fig. 3 A detailed overview of the risk factors with the respective ICD and OPS codes as well as confidence intervals, Odds Ratios and p-values can be found in a summary table in the Additional file 1: Table S1.

### Predictive quality of the model

**Fig. 4:**
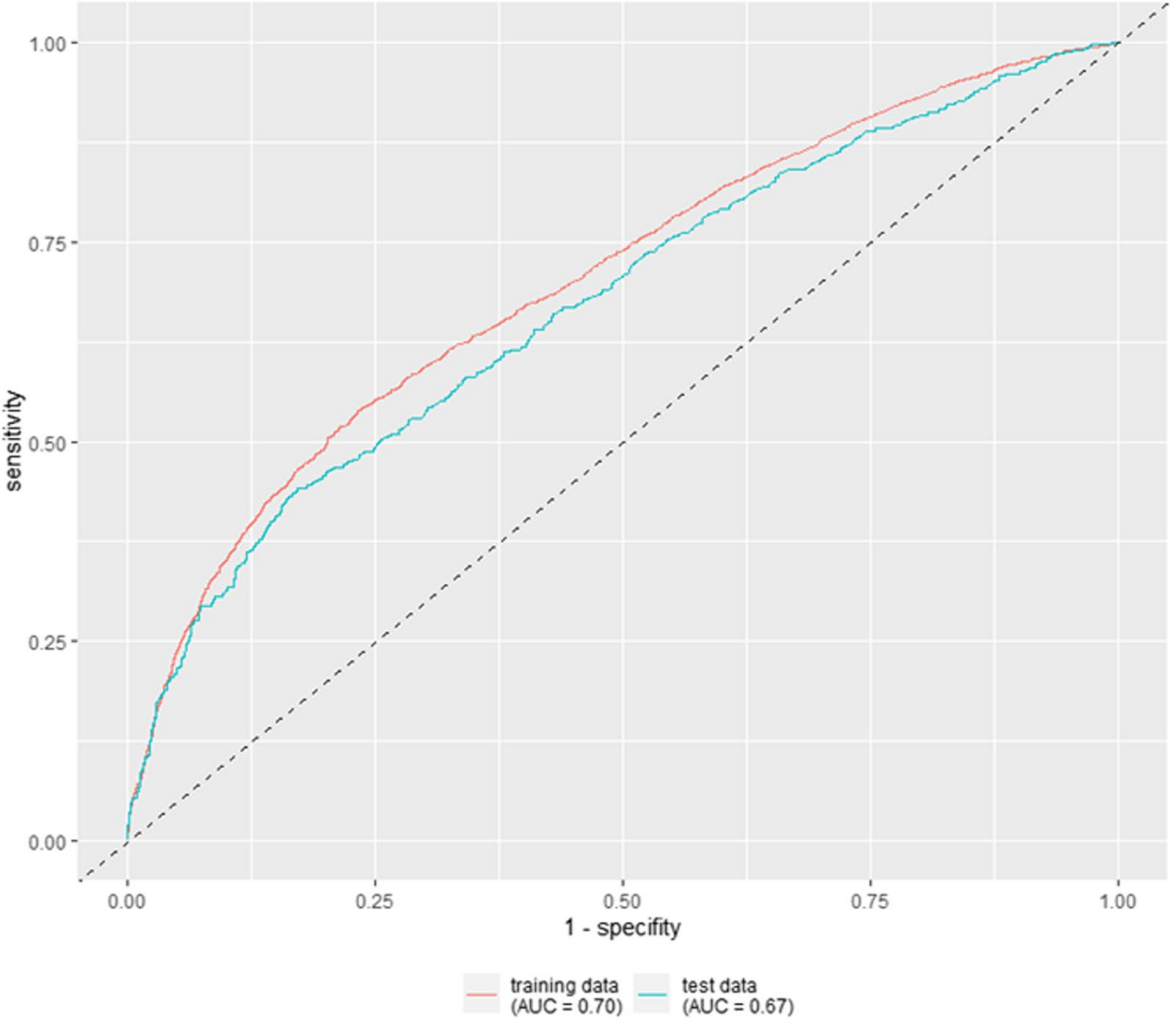
Receiver operating characteristic (ROC) curves for training and test data. The figure shows the ROC curves of the model for the training data set (red) and the test data set (blue). *ROC* receiver operating characteristic, *AUC* area under the curve

ROC analyses were used to assess the diagnostic value of the combination of all the factors that had been found to be predictive in the previous analysis; the c-value on the data set used for creating the model is 0.700 and can be classified as an acceptable predictive value according to Hosmer and Lemeshow [10]. The prognostic model was validated using an independent test data set from 2018. The patient data of the training data set and those of the test data set showed no relevant differences in terms of invasive long-term ventilation, in-hospital mortality rate and ventilation hours, see Table 3. The AUC value for the test dataset was c = 0.679, with a sensitivity and specificity of 49 and 80% respectively (when classifying patients based on their predicted probability with a cut-off of 41.15%), which is slightly lower than the result based on the original data. We conducted for e.g. a Hosmer and Lemeshow Goodness-of-Fit Test (DF 8, Chi^2^ 8.86) with a p-value of 0.35 and therefore our model fits the data. The ROC curves for the training and the test data are shown in Fig. 4.

**Table 3:** Patients characteristics

		Training data (2015–2017)	Validation data (2018)
Hospitalisations		7758	2.031
Patients		7207	1923
Female	%	37.2	38.0
Age	Mean (SD)	69.8 (11.3)	70.3 (11.1)
Invasive long-term ventilation	%	39.0	36.6
mortality rate in hospital	%	25.2	26.1
hours of ventilation	Mean (SD)	429.6 (386.7)	403.8 (331.7)

The table shows the characteristics of the patients in the training and test datasets

## Discussion

In the face of increasingly complex intensive care interventions and an aging population, the prevention of long-term IMV is one of the great challenges in modern intensive care medicine. Our aim was to identify risk factors for long-term ventilation early after initiation of invasive mechanical ventilation. Using data from more than 7,500 inpatient hospitalisations, we identified a set of risk factors that can be assessed in the first 96 h after intubation. Drawing on the knowledge of a multi-professional advisory team and a systematic literature review [8], the use of claims data enabled an exploratory research approach that allowed us to analyse a wide range of different diagnoses. By systematically screening all three-digit ICD codes and selected four- and five-digit ICD codes, we considered more than 1,400 different diagnoses in the analyses. Through this approach, several previously unknown risk factors as well as favourable conditions for a subsequent invasive long-term IMV have been identified. Compared to the large current WEAN SAFE study, which was designed to understand the weaning process in a large, realistic population of intensive care patients who have been on IMV for at least 48 h and are therefore at risk of prolonged weaning and weaning failure, our study targets a similar patient population, but not the identical one. The patients we studied were thought to have a slightly higher risk of weaning failure with subsequent long-term ventilation, were over 30 years of age, had at least one medical comorbidity and were on invasive ventilation for at least 96 h [6]. In contrast with the literature [2, 6, 11, 12], age did not play a role in our analysis. This finding is surprising, as age was also a relevant factor in the WEAN SAFE study [6], the most up-to-date and comprehensive analysis on this subject. Our finding may be explained by the fact that the nature of our analysis allowed us to consider a wide range of pre-existing conditions and procedures, which reflect the individual level of comorbidity relatively well. It is likely that age is associated with a higher risk of having multiple comorbidities, which in turn are associated with an increased risk of weaning failure, but is not a risk factor in itself. As outlined in previous studies, COPD [2, 12, 13], evidence of previous dependence on a ventilator invasive or non-invasive [2, 14], colonisation with multidrug-resistant pathogens [15] and cerebral infarction [16] were confirmed as risk factors in our study population. Other risk factors not previously described include medical history, preexisting conditions, admission diagnoses, resource prescriptions, and procedures performed within the first 96 h after initiation of IMV.

Unexpectedly, nursing home accommodation immediately before hospitalisation was a prognostically favourable factor. This can best be explained by the fact that the treating intensivists performed a thorough pre-selection of these patients with regard to the general prognosis before initiating IMV. Furthermore, nursing home accommodation is associated with more continuity of care and social support than living at home (e.g. also for widowers). Therefore, the care after hospital stay is better. A limitation of claims data is that information on nursing care and social support is limited/absent for those who live at home. Among admission diagnoses, thyroiditis, eating disorders, rheumatic mitral valve disease and acute pancreatitis were identified as risk factors for subsequent long term IMV. In the case of thyroiditis, hypothyroidism, which often develops during the course of the disease, probably plays a role. However, both hyperthyroidism [17] and hypothyroidism [15, 18] can affect respiratory function through muscle weakness. In the context of eating disorders, in addition to general cachexia-related muscle weakness, hypophosphataemia [19] may also be taken into account, as indicated by a small study on the effect of serum phosphorus concentration on ventilatory weaning [20]. Rheumatic mitral valve disease is the most common valvular heart disease [21] and can lead to heart failure via tricuspid regurgitation, which in turn is a known risk factor for long-term ventilation [21]. Pre-existing dementia and previous placement of a dialysis fistula, as well as peritonitis, cardiac arrhythmias, and pulmonary or abdominal metastases as admission diagnoses turned out to be favourable factors with respect to subsequent long-term ventilation. The association of known dementia with delirium [22] in the context of acute hospitalisation may have led to the administration of more sedative medications and, via this, to an increase in the duration of ventilation, but without requiring subsequent long-term ventilation. In the case of the above-mentioned admission diagnoses, both cardiac arrhythmias and peritonitis are causally treatable diseases, which allows termination of ventilation after successful completion of treatment. The same is true for patients with a dialysis fistula; here, the likely pathogenesis of respiratory failure is volume overload, which can be rapidly corrected. With regard to metastases and dementia, we assume a selection bias; usually, only patients with a very favourable prognosis are admitted to an intensive care unit in this situation [23]. Of the operations and procedures studied within the first 96 h initiation of IMV, particularly procedures that indicate a pulmonary cause of the need for ventilation, such as bronchoscopy or computed tomography of the chest, were found to be risk factors for long-term IMV. Also, early tracheostomy, which was associated with a very high risk, is certainly an indicator that the treating physicians already apprehended prolonged weaning. Patients with particularly complex prolonged ICU courses were also at increased risk for long-term ventilation, indicative of the use of extracorporeal life support (ECLS), positioning therapy, transfusion of plasma components or coagulation factors. The use of a chest tube as a further risk factor indicates either a pre-existing pulmonary condition or complications related to barotrauma or iatrogenesis [24]. In addition, procedures suggestive of leading neurological problems such as cerebral spinal surgeries, cerebrospinal fluid system surgeries, or cranial imaging were also predictors for unfavourable outcomes. In contrast, radical cervical lymphadenectomy, or autologous blood collection and transfusion, usually as part of elective surgery, showed a favourable prognosis with respect to subsequent long-term ventilation. The combination of all identified risk factors makes it possible to assess the prognosis with regard to subsequent long-term ventilation in the first days of intensive medical care with an acceptable predictive accuracy. The predictive value of this model, could be confirmed based on a subsequent validation cohort. Strengths of this model are the large number of patients, the validation in a later cohort and the 30-day follow-up. Despite the steadily increasing number of long-term ventilated patients, the associated individual suffering and the high costs for the health care systems, there are only a few studies that have dealt with the determination of risk factors of invasive ventilation [8]. One of the large studies on ventilatory weaning by Béduneau et al., the WIND study, provides a multicentre population of 2729 ventilated patients and identified age, Sequential Organ Failure Assessment (SOFA) Score at admission, duration of IMV before the first separation attempt and medical admissions as risk factors for weaning failure. However, the study did not aim to identify risk factors but to describe the weaning process, according to a new operational classification [11]. Two smaller studies from China with 302 and 343 patients investigated risk factors for prolonged mechanical ventilation and weaning failure and found age > 74 years and COPD as well as Glasgow Score and PaCO_2_ (at the beginning of the first spontaneous breathing trial) as risk factors [13, 25]. The most comprehensive study dealing with weaning failure is the study by Windisch et al. It is a retrospective analysis of a German weaning registry, here the data of 11,424 patients transferred to a specialised weaning centre were examined, the need to continue with invasive ventilation was most strongly associated with the duration mechanical ventilation prior to transfer from the ICU, a low body mass index, pre-existing neuromuscular disorders and advanced age [2].

The current WEAN SAFE study also examined factors associated with weaning failure. Demographic factors independently associated with weaning failure included older age, weakened immune system and frailty. Critical illness-related factors associated with weaning failure were severity of critical illness as measured by the SOFA score, cardiac arrest or a non-traumatic neurological event as the reason for admission to the ICU, pre-existing limitations of care, and the degree of respiratory dysfunction (respiratory rate and lower partial pressure of arterial oxygen relative to FiO_2_, fraction of inspiratory oxygen) and ventilatory support (dynamic driving pressure and PEEP, positive end expiratory pressure) used at the time of the first separation attempt. Among the potentially modifiable factors, the presence of deep sedation levels at the time of the first weaning attempt was associated with weaning failure; and the time interval between the development of weaning criteria and the first weaning attempt was independently associated with weaning failure [6]. In addition to consistent and timely implementation of weaning attempts when weaning criteria are met, we need models that allow us to identify high-risk patients as early as possible.

### Limitations and advantages

The main drawback of our study includes the use of data on health care services designed for reimbursement with all the associated problems [26]. A possible selection bias lies in the fact that the data of a single health insurance fund from a single federal state in Germany was analysed. Although the AOK is the largest health insurance fund in Germany, distortion effects cannot be ruled out due to the special structure of the insured population compared to other health insurance funds, particularly those with private insurance. Further possible problems arise from regional peculiarities, as only the region of Baden Württemberg was examined in the analyses.

Additionally, specific issues related to research question may not be integrated in the coding of the DRG-system and that identical coding does not necessarily mean identical clinical handling and assessing. However, the clear advantages are the comparability and standardisation of the data also including the predefined definitions. Another advantage is that this approach images the clinical reality in so far as not only specific or academic hospitals are involved in the data acquisition, but all other hospitals as well. With regard to the chosen time points for follow-up, we had to rely on data that could be obtained from the health insurance records. In particular, the choice of follow-up periods of IMV ≥ 500 h or readmission with IMV within 30 days after the first discharge from the hospital could be too short an endpoint. In terms of comparability with other clinical trials, day 28 as the usual ICU endpoint and readmission within 90 days might have been better. Claims data analysis provides an opportunity to comprehensively study a specific patient group that is difficult to include in prospective clinical trials in a large patient population to gain additional information. A lot of clinically relevant information, such as the SOFA score, laboratory values and blood gas analysis, as well as mechanical ventilation parameters, cannot be captured in such an analysis. The results should be seen as complementary to clinical trials, which offer the opportunity to investigate the associations shown here in more detail in smaller populations.

## Conclusions

Despite methodological biases, the risk of long-term ventilation could be determined early in the ventilatory course based on health claims data alone. We expect that the prediction quality can be further improved by combining the existing model with additional clinical information, such as neurological status, respirator settings, breathing mechanics, blood gas parameters and other biomarkers. Whether the application of this model is useful in clinical practice and whether it can contribute to better care for invasive patients is currently being investigated in the multicentre PRiVENT project.

### Supplementary Information

**Additional file 1: Table S1.** Odds ratios of predictors.

## Data Availability

The datasets used and/or analysed during the current study are available from the corresponding author on reasonable request.

## Abbreviations

aQua: Institute for Applied Quality Improvement and Research in Health Care
AOK-BW: *Allgemeine Ortskrankenkasse Baden-Württemberg*
CI: Confidence interval
COPD: Chronic obstructive pulmonary disease
ECLS: Extracorporeal life support
ECMO: Extracorporeal membrane oxygenation
FiO_2_: Fraction of inspiratory oxygen
HMV: Home mechanical ventilation
ICD: International Statistical Classification of Diseases and Related Health Problems
ICU: Intensive care unit
IMV: Invasive mechanical ventilation
OPS: Operation and procedure key (German “Schlüssel”)
ROC: Receiver operating characteristic
PEEP: Positive end exspiratory pressure
SOFA: Sequential Organ Failure Assessment
STROBE: Strengthening the Reporting of Observational Studies in Epidemiology
VIF: Variance inflation factor
