# Supplementary material for: Risk factors for long-term invasive mechanical ventilation: a longitudinal study using German health claims data
Source: Respir Res. 2024 Jan 27;25:60. doi: 10.1186/s12931-024-02693-6 (PMC10821552; doi:10.1186/s12931-024-02693-6)
Supplement: Supplementary file 1 — Additional file 1: Table S1. Odds ratios of predictors. [file 12931_2024_2693_MOESM1_ESM.docx]

| Predictor | Definition | Odds Rato | | | Lower CI | Upper CI | P value |
| --- | --- | --- | --- | --- | --- | --- | --- |
| Baseline predictors |  | **1.56** | | | **1.05** | **2.32** |  |
| Intercept |  | 0.32 | | | 0.22 | 0.46 | < 0.001 |
| Age (years) |  | 1.00 | | | 1.00 | 1.01 | 0.276 |
| Gender (female) |  | 0.93 | | | 0.84 | 1.03 | 0.149 |
| Nursing home accommodation | Nursing home accommodation  immediately before hospitalisation | 0.67 | | | 0.50 | 0.90 | 0.009 |
| Diagnoses | |  | | |  |  |  |
| *Pre-existing medical conditions (in 365 days prior to the ventilator case).* | |  | | |  |  |  |
| Thyroiditis | ICD E06 | 1.50 | | | 1.01 | 2.22 | 0.045 |
| Dementia | ICD F00-F03 | 0.66 | | | 0.55 | 0.80 | < 0.001 |
| Eating disorders | ICD F50 | 1.99 | | | 1.09 | 3.65 | 0.026 |
| Rheumatic mitral valve disease (insufficiency or stenosis) | ICD I05 | 1.89 | | | 1.35 | 2.65 | < 0.001 |
| Peritonitis | ICD K65 | 0.45 | | | 0.26 | 0.78 | 0.004 |
| Pneumothorax | ICD J93 | 2.10 | | | 1.12 | 3.93 | 0.020 |
| *Admission diagnosis* | |  | | |  |  |  |
| Cardiac arrhythmia | ICD I49 | 0.51 | | | 0.31 | 0.86 | 0.011 |
| Cerebral infarction | ICD I63 | 1.54 | | | 1.17 | 2.03 | 0.002 |
| Acute pancreatitis | ICD K85 | 2.64 | | | 1.50 | 4.63 | 0.001 |
| *Diseases (previous disease, admission diagnosis, ventilation case).* | |  | | |  |  |  |
| Dependence (at least 3 completed months) on aspirator and/or respirator. | ICD Z99.0, Z99.1 | 5.13 | | | 4.03 | 6.52 | < 0.001 |
| COPD | ICD J44 | 1.30 | | | 1.17 | 1.44 | < 0.001 |
| Pulmonary or abdominal metastasis | ICD C78 | 0.49 | | | 0.35 | 0.68 | < 0.001 |
| Operations and procedures | |  | | |  |  |  |
| *Operations and procedures in the 365 days prior to the ventilator case.* | |  | | |  |  |  |
| Tracheostomy, permanent or temporary | OPS 5311, 5312 | 2.17 | | | 1.67 | 2.82 | < 0.001 |
| Creation of a dialysis fistula, shunt or bypass | OPS 5393 | 0.36 | | | 0.18 | 0.72 | 0.003 |
| *Operations and procedures during the ventilation case up to 95h after intubation.* | | | | | | | |
| Bronchoscopy | OPS 1620 | | 1.18 | 1.07 | | 1.31 | 0.001 |
| Native computed tomography of the chest | OPS 3202 | | 1.19 | 1.03 | | 1.37 | 0.019 |
| Computed tomography and/or magnetic resonance imaging of the the cranium with imaging contrast medium | OPS 3220, 3820 | | 1.36 | 1.16 | | 1.59 | < 0.001 |
| Operations on the spinal cerebrospinal fluid system (drainage, shunt, catheter; also, removal) | OPS 5038 | | 2.61 | 1.31 | | 5.22 | 0.007 |
| Tracheostomy, permanent or temporary | OPS 5311, 5312 | | 3.97 | 3.43 | | 4.60 | < 0.001 |
| Radical cervical lymphadenectomy | OPS 5403 | | 0.20 | 0.10 | | 0.40 | < 0.001 |
| Chest tube | OPS 8144 | | 1.38 | 1.18 | | 1.62 | < 0.001 |
| Positioning treatment in a special bed (e.g. positioning in a rotating or sandwich bed) | OPS 8390.0 | | 2.31 | 1.70 | | 3.13 | < 0.001 |
| Autologous blood collection and transfusion | OPS 8803 | | 0.65 | 0.47 | | 0.90 | 0.010 |
| Transfusion of plasma components and genetically engineered plasma proteins | OPS 8810 | | 1.36 | 1.17 | | 1.58 | < 0.001 |
| PECLA, ECCO2R, vv- und va ECMO und Pre-ECMO therapy | OPS 8852 | | 1.80 | 1.35 | | 2.39 | < 0.001 |
| Complex treatment for colonization or infection with multidrug-resistant pathogens | OPS 8987 | | 1.49 | 1.21 | | 1.84 | < 0.001 |

**Table S1: Odds Ratios of predictors**

The table shows all predictors of the model with the respective odds ratios and the corresponding confidence intervals. In addition to the master data, diagnoses as well as operations and procedures of the previous 365 days, before the intensive care stay and operations and procedures during the hospital stay up to 95 h after intubation were considered. Abbreviations: COPD chronic obstructive pulmonary disease, PECLA pumpless extracorporeal lung assist, ECCO2R Extracorporeal CO2 removal, vv veno-venous, va veno-arterial, ECMO extracorporeal membrane oxygenation, CI confidence interval.
